# What Has Been Seen Cannot Be Unseen—Detecting Auxin In Vivo

**DOI:** 10.3390/ijms18122736

**Published:** 2017-12-16

**Authors:** Barbora Pařízková, Markéta Pernisová, Ondřej Novák

**Affiliations:** 1 Laboratory of Growth Regulators, Centre of the Region Haná for Biotechnological and Agricultural Research, Faculty of Science of Palacký University & Institute of Experimental Botany of the Czech Academy of Sciences, Šlechtitelů 27, CZ-783 71 Olomouc, Czech Republic; barbora.parizkova@upol.cz; 2 Department of Chemical Biology and Genetics, Centre of the Region Haná for Biotechnological and Agricultural Research, Faculty of Science of Palacký University, Šlechtitelů 27, CZ-783 71 Olomouc, Czech Republic; pernisov@sci.muni.cz; 3 Functional Genomics and Proteomics, National Centre for Biomolecular Research, Faculty of Science, Masaryk University, Kamenice 5, CZ-62500 Brno, Czech Republic

**Keywords:** auxin, auxin signalling, auxin distribution, auxin transport, indirect visualization, direct visualization, receptor, sensor

## Abstract

Auxins mediate various processes that are involved in plant growth and development in response to specific environmental conditions. Its proper spatio-temporal distribution that is driven by polar auxin transport machinery plays a crucial role in the wide range of auxins physiological effects. Numbers of approaches have been developed to either directly or indirectly monitor auxin distribution in vivo in order to elucidate the basis of its precise regulation. Herein, we provide an updated list of valuable techniques used for monitoring auxins in plants, with their utilities and limitations. Because the spatial and temporal resolutions of the presented approaches are different, their combination may provide a comprehensive outcome of auxin distribution in diverse developmental processes.

## 1. Introduction

Auxin, which was the first-identified plant hormone, plays a fundamental role in plant growth and development (e.g., inducing vascular tissue differentiation, tropic responses, and promoting root development). Indole-3-acetic acid (IAA) is the main natural auxin, but some plants contain other compounds that display weak auxin activity (e.g., phenylacetic acid). Several synthetic auxins (e.g., 1-naphthaleneacetic acid, 1-NAA) are often used in commercial applications [1]. The functionality of all components of auxin signalling and homeostasis is essential for proper plant development.

The cellular presence of an endogenous or exogenous (e.g., synthetic) auxin is perceived by the TRANSPORT INHIBITOR RESPONSE1/AUXIN SIGNALING F-BOX (TIR1/AFB) signalling pathway, and triggers the expression of the target genes, which induce biological responses to the received stimulus. The auxin signalling TIR1/AFB pathway comprises three major families of proteins: (i) auxin nuclear receptor TIR1/AFB F-box proteins; (ii) AUXIN RESPONSE FACTOR (ARF) transcription factors; and, (iii) AUXIN/INDOLE 3-ACETIC ACID INDUCIBLE (Aux/IAA) repressor proteins [2]. In the absence of auxin, Aux/IAAs bind ARF transcription factors disabling their function. Auxin binding to TIR1/AFB induces the proteasomal-dependent degradation of Aux/IAA by targeting the domain II for ubiquitination, and thus releases ARFs from repression enabling auxin response.

While the TIR1/AFB signalling pathway is fully explained at the molecular level [3], the function of other factors playing a role in response to auxin stimuli has not yet been fully understood [4]. These factors include, for example, (i) the S-PHASE KINASE-ASSOCIATED PROTEIN 2A (SKP2A) protein that could bind auxins in order to regulate cell division; (ii) SMALL AUXIN UP RNA (SAUR) proteins that are likely involved in cell elongation; (iii) INDOLE 3-BUTYRIC ACID RESPONSE 5 (IBR5); and, (iv) AUXIN BINDING PROTEIN 1 (ABP1), which is the oldest known putative auxin receptor [5,6]; however, these latest findings were put into question when ABP1 was found to have little, if none, prominent role in *Arabidopsis* development [7].

The differential and dynamic distribution of auxins within individual plant tissues depends on auxin homeostasis (metabolism and transport). Free auxin levels are maintained by its metabolism (biosynthesis, conjugation, and degradation), which occur predominantly in rapidly growing meristematic areas or organs, such as a shoot tip, a root tip, or emerging leaves [8]. The IAA is de novo synthesised through two biosynthetic pathways: (i) l-tryptophan (l-Trp) independent, well described in microorganisms [9]; and (ii) Trp-dependent, which includes four biosynthetic pathways that are named according to their first intermediates, and which is a significant source of endogenous IAA for higher plants [10]. The auxin metabolism comprises (i) an oxidative catabolism leading to the inactive 2-oxindole-3-yl acetic acid (oxIAA) [11]; and, (ii) a conjugation with sugars, amino acids, peptides, or proteins [12]. IAA conjugates have transport, storage, and deactivation functions, which ensure the maintenance of auxin homeostasis [13].

Another important process that is involved in the concentration gradient is passive and active auxin transport. In higher plants, auxins are transported together with assimilates through the vascular system at long distances (phloem). At a short distance (cell-to-cell), a polar active movement combines the chemiosmotic force, ATP hydrolysis and auxin transporters [14]. Major protein carriers that are present in the auxin transport are (i) AUXIN RESISTANT 1 (AUX1) and LIKE-AUX1 (LAX) from the subfamily of amino acid permeases contributing to auxin influx [15]; (ii) PIN-FORMED (PIN) transmembrane proteins specifically delivering auxin molecules out of the cell and regulating intracellular auxin homeostasis [16]; (iii) P-GLYCOPROTEINS/ATP-BINDING CASSETTE SUBFAMILY B (PGP/ABCB) transmembrane transporters [17]; and, (iv) PIN-LIKES (PILS) proteins with structural similarity to PIN proteins that are localised in the membrane of the endoplasmic reticulum [18]. The loss of the asymmetric distribution of auxin due to the genetic alteration of PIN function affects many developmental processes, e.g., embryogenesis, organogenesis, tissue differentiation, and various tropisms [19,20,21]. It has also been shown several times that auxin influx carriers (AUX1/LAX) play an important role during gravitropism, phototropism, lateral root, and root-hair development [22,23,24,25]. Furthermore, additional substances, such as flavonols, have been recently proposed as endogenous auxin transport regulators [26,27]. Flavonols are plant phenolic secondary metabolites that have been suggested as auxin transport inhibitors [28]. Based on the fact that auxin transport is elevated in the absence of flavonoids is and reduced in the presence of excess flavonols, they are thought to act as auxin efflux modulators [29] that are targeting both PIN [30,31] and ABCB [32,33] auxin efflux facilitators. Nevertheless, the regulation of auxin distribution by flavonols seems to be more complex, involving auxin signalling [34,35], changes in vesicular trafficking [31], or protein phosphorylation [36].

In this review, we focus on the indirect and direct methods for visualization of auxin signalling, metabolism, and transport. We describe the recent advances in monitoring auxin distribution and signalling, as well as bioanalytical tools for the quantification and visualisation of auxin metabolites at tissue and cellular level.

## 2. Indirect Auxin Visualization—Methods Based on Detection of Auxin Action

### 2.1. Reporters Based on Auxin Signalling

Visualisation of auxin in plants, direct or indirect, has attracted a lot of interest in phytohormone research for many years. The first auxin reporters were made of promoters of auxin inducible genes that were fused to a *β-glucuronidase (GUS)* reporter gene, such as *SAUR:GUS* transformed into tobacco [37] or soybean GRETCHEN HAGEN 3 (GH3)-derived *GH3:GUS* used in white clover (*Trifolium repens*) [38]. Both of the reporters were able to show an asymmetric pattern of the auxin action during gravitropism or phototropism.

Going more into details on the DNA sequence, a 183-bp auxin-responsive region (AuxRR) of the *PsIAA4/5* promoter was identified in *Pisum sativum* containing two auxin-responsive domains (AuxRD) A and B defined by linker scanning mutagenesis [39,40]. AuxRD A possesses a conserved sequence ^T^/_G_GTCCCAT and has been described as an auxin switch, while AuxRD B was hypothesised to have an enhancer-like activity, with ^C^/_A_ACATGGN^C^/_A_^A^/_G_TGT^T^/_C_^T^/_C_^C^/_A_ nucleotide sequence [39]. Domains A and B were cloned to control *GUS* expression in a *BA:GUS* construct and tested in *Arabidopsis* for their functionality [41]. In the root elongation zone, the expression of *BA:GUS* was induced by active auxins such as IAA, NAA or 2,4-dichlorophenoxyacetic acid (2,4-D); and, less by indole-3-butyric acid (IBA). Moreover, other tested compounds, such as inactive auxin analogue, IAA metabolic precursors, IAA transport inhibitors, or phytohormones, were unable to induce *GUS* expression. *In planta,* the inducibility of the *BA:GUS* reporter gene by IAA was increased from 10^−7^ M to 10^−4^ M, but was inhibited at 10^−3^ M. In addition, *BA:GUS* expression pattern was confirmed by introducing the second reporter gene, encoding the green fluorescent protein (GFP), under the control of BA sequence. *BA:GFP* expression displayed a similar pattern to that of *BA:GUS*, and was inducible by auxin as well [41]. Using chemical genetics in *Arabidopsis*, *BA:GUS* reporter has been successfully used as bait for the identification of inhibitors of auxin transcriptional activation [42].

#### 2.1.1. The Signalling Reporter *DR5* and Variants

**Figure 1:**
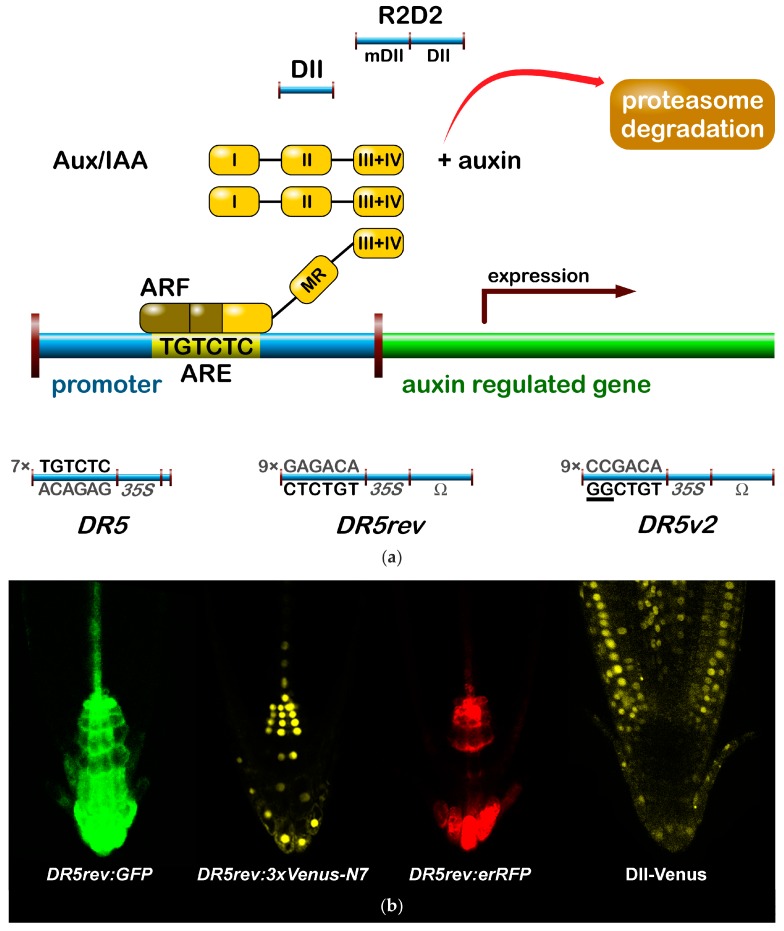
Indirect auxin reporters. (**a**) *DR5* reporters were derived from auxin response element (ARE) sequence for binding of ARF transcription factors in auxin responsive promoters. (**b**) The expression of *DR5rev:GFP*, *DR5rev:3xVenus-N7* and *DR5rev:erRFP* reflects similar auxin signalling output in *Arabidopsis* root tip. Degradation based reporters DII and R2D2 contain degron domain from Aux/IAA repressors leading to ubiquitination and degradation in the presence of auxin. They represent auxin signalling input. *35S*, *CaMV35S* minimal promoter; ARE, auxin response element; ARF, AUXIN RESPONSE FACTOR; Aux/IAA, AUXIN/INDOLE-3-ACETIC ACID; GFP, green fluorescent protein; RFP, red fluorescent protein; Venus, yellow fluorescent protein; and, Ω, *tobacco mosaic virus* leader sequence.

The most popular auxin reporter to indirectly visualise auxin in plants is the artificial auxin-response promoter *DR5* [43], whose activity reflects an auxin response maximum [44]. Among several auxin inducible genes, *GH3* from a soybean was identified as rapidly and specifically induced by auxins [45]. Transcriptional activation of this gene was observed within 5 min after auxin application [46]. Within the *GH3* promoter, the smallest composite natural auxin response element (AuxRE) with strict auxin specificity was identified and named *D1-4* element [47]. The *D1-4* represents an 11 bp 5′-CCTCGTGTCTC-3′ sequence, and contains a coupling element that overlaps with the TGTCTC motif required for auxin inducibility [48]. The TGTCTC sequence occurs in many promoters of early auxin responsive genes, bound by ARFs and responding rapidly to active auxins only [47] (Figure 1a). Together with the GGTCCCAT sequence that was identified in a pea [39], it is also present as a TGTCTCtcatttGGTCCCAT sequence in *SAUR* promoters [49].

Thymidine substitutions in the natural *D1-4* AuxRE (CCTCGTGTCTC) provided the synthetic *DR5* AuxRE 5′-CCTttTGTCTC-3′, with an exceptionally strong auxin response when cloned upstream of a minimal −46 *cauliflower mosaic virus (CaMV) 35S* promoter [43]. Eight repeats of the synthetic *DR5* (8x) AuxRE displayed up to 10-fold higher inducibility by NAA when compared with the eight repeats of natural *D1-4* (8x) AuxRE. In addition, the spacing between TGTCTC elements and nucleotide composition upstream of TGTCTC elements was suggested to be important for the auxin inducibility in the *DR5* construct [43]. Several variants of *DR5* element were prepared to monitor auxin signalling action in plants (Figure 1). Seven tandem repeats of the 11 bp sequence 5′-CCTTTTGTCTC-3′ fused to a −46 bp *CaMV35S* minimal promoter and driving the *GUS* gene gave a rise to the *DR5:GUS* reporter [50]. Nine inverted repeats of the 11 bp element, a *CaMV35S* minimal promoter and a *TMV* leader sequence were used to create a *DR5rev* version of the auxin responsive promoter. Different reporter genes were combined with *DR5rev* promoter, such as phosphonate monoester hydrolase *PEH A* gene in *DR5rev:PEHA* [51], an endoplasmic reticulum-targeted green fluorescent protein in *DR5rev:GFP* [44] (Figure 1b), three tandem copies of Venus, a fast maturating variant of the yellow fluorescent protein, fused to a nuclear localization signal (NLS) in *DR5rev:3xVenus-N7* [52] (Figure 1b), a red fluorescent protein (RFP) targeted to the endoplasmic reticulum in *DR5rev:mRFPer* [53] and *DR5rev:erRFP* [54] (Figure 1b), or a luciferase coding region in *DR5:Luciferase* [55]. Overall, transgenic *Arabidopsis* plants that were carrying these reporters displayed a similar pattern, with visible staining in root quiescent centre (QC), columella cells, protoxylem, the most distal domain of developing shoot primordia with an incipient leaf vein and in root primordia tips. It has been shown that the activity of *DR5* correlates with auxin accumulation detected by immunolocalisation in *Arabidopsis* [56].

To create a more sensitive auxin responsive promoter, two bases in the original *DR5* binding sequence TGTCTC were exchanged to make a TGTCGG with higher binding affinity to ARF, as identified by protein binding microarrays [57]. Interestingly, the TGTCGG sequence occurs also in a promoter of *Agrobacterium tumefaciens* T-DNA of *Ach5 Ti* plasmid [58]. Nine original AuxREs in the *DR5rev* promoter were replaced with new binding site elements producing a *DR5v2* promoter [59] (Figure 1a). The expression pattern of *DR5v2* matches more precisely the auxin accumulation sites, as predicted from the localisation of the polar auxin transporters [60]. Moreover, *DR5v2* showed a weak activity in the dividing cells of the embryo, leaf, or shoot meristem corresponding to an auxin function in cell division processes [61]. When comparing the activity of *DR5* and *DR5v2* in a *DR5v2:ntdTomato-DR5:n3EGFP* double reporter [59], all of the expression sites of *DR5* were overlapped by a *DR5v2* expression and the additional *DR5v2* signal appeared in other cell types (cotyledons and vasculature during embryogenesis, in metaxylem, pericycle, lateral root cap, epidermal cells of root, and in the cells surrounding the shoot primordia and the L1 layer of the shoot apical meristem). The difference in *DR5* and *DR5v2* sensitivity and localisation can be useful for the identification of unique regulatory factors, preferring specific AuxRE binding sequences in both promoters.

#### 2.1.2. Degradation-Based Auxin Reporters

In addition to *DR5*, another type of auxin responsive promoter was constructed to monitor auxin signalling input [62]. The auxin interacting domain II (DII) [63] of IAA28 protein was cloned under a constitutive promoter and was fused to Venus with a NLS sequence [64] to generate the DII-Venus auxin sensor (Figure 1). The DII domain is the Aux/IAA domain that is ubiquitinated and induces degradation of the protein in response to the auxin dose-dependent presence. Therefore, DII-Venus monitors the input into the auxin signalling pathway by the degradation of fusion protein, thus switching off the signal in the presence of auxin, in an opposite manner to *DR5* principle. Two promoter variants were used for the sensor: a *CaMV35S* promoter [64] or a *RPS5A* promoter [59]. The need of “auxin input” quantification led to the development of an innovated reporter. The combination of DII-Venus and mDII-ntdTomato, a mutated auxin insensitive variant of DII, into one construct gave a rise to the ratiometric version of the auxin input—R2D2 [59] (Figure 1a). Two fluorophores allow for a semiquantitative measurement of auxin accumulation as a ratio of yellow and red signal. Auxin sensitive DII and R2D2 reporters enable the observation of fast changes in auxin accumulation at cellular resolution in real-time [59,64,65,66,67]. Based on DII degradation, another quantitative ratiometric sensor for analysis of auxin dynamics in real-time was developed and optimised for the use in single cell systems combining a luminescent reporter with an internal normalization element [68].

Interestingly, DII and R2D2 reporters showed partial auxin insensitivity in the root tip, particularly in the epidermis, cortex, and endodermis cell files that are close to the QC [59,64,65]. After the gravistimulation or exogenous auxin application, the DII-Venus signal of both the reporters is not switched off completely in these cells, suggesting a distinct type of regulation when compared to cells without signal. Moreover, the comparison of *DR5* and DII signals revealed discrepancies between the auxin signalling response input and output, suggesting the presence of the auxin, but the absence of a signalling response in particular parts of the growing plant [59]. It would be useful to combine *DR5v2* and R2D2 in a single three-colour reporter to inspect the auxin input and output in one plant.

#### 2.1.3. Dissecting the Specificity of the Auxin Signalling

To follow the specificity of the auxin signalling, a set of Aux/IAA and ARF reporters were fused with *GUS* or *GFP* tag to report signalling pathways with particular sets of Aux/IAA and ARF proteins. An ARF collection using transcriptional fusion with nuclear localised *3xGFP* mapped their different, as well as overlapping expression pattern in embryo and in the root tip [69]. Analogically, members of Aux/IAA family possess a wide range of localization patterns in *Arabidopsis*, suggesting their spatiotemporal specificity [70,71,72,73,74,75,76,77,78]. When combining the members of Aux/IAA and/or ARF families provides a huge set of possible mutual interactions pointing to variability and complexity of the auxin signalling in plant development [62,79] and waiting to be revealed.

### 2.2. Focused on Auxin Source

Inspecting auxin production by the activity of auxin biosynthetic genes provides us another approach how to visualize auxin indirectly. Indeed, auxin biosynthesis pathways are represented by a wide scale of participating enzymes [80]. Several biosynthetic pathways produce free IAA most probably in the tissue-, cell-, or time-dependent manner, reflecting plant development plasticity and adaptability. The expression patterns of two related enzymes in the probably essential Trp-dependent auxin biosynthetic pathway, *TAA1p:GFP-TAA1* (*TRYPTOPHAN AMINOTRANSFERASE OF ARABIDOPSIS 1*), and *TAR2p:GUS* (*TRYPTOPHAN AMINOTRANSFERASE RELATED 2*), are complementary in stele, QC, and columella cells [81]. Subsequent enzymatic step to produce IAA is catalysed by flavin-containing monooxygenases from the YUCCA (YUC) family. Several fusion variants of the *YUC1* to *YUC11* to a *GUS*, nuclear-targeted *3xGFP*, or a cytosolic *GFP-GUS* tag showed auxin production specificity in flower organs [82], during embryo development [83,84] and leaf formation [83], or in the root tip [85]. The expression patterns of these genes point to the root meristem as a very active place for auxin biosynthesis [81,85].

### 2.3. Following Auxin Flow

**Figure 2:**
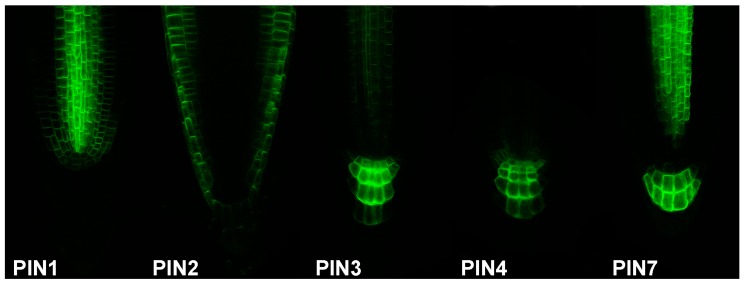
Visualisation of auxin flow. Functional translational fusion of auxin transport proteins enables to predict auxin distribution in *Arabidopsis* root tip. Auxin efflux carriers from the PIN family were fused with GFP. PIN, PIN-FORMED.

Auxin biosynthesis reporters in combination with the reporters of the auxin transport machinery mark the auxin source and subsequent auxin flow. As auxin efflux carriers from the PIN family represent limiting factors of auxin transport [86], they can serve as an arrow of auxin flow direction by their polar cell localisation, and sites with high auxin concentration can be therefore predicted. Grouped by their structure [87], “long” PINs (1–4, 7) enable intercellular auxin transport with partially redundant function [88,89], while “short” PINs (5, 6, 8) participate mainly in intracellular auxin distribution. Over the years, an almost complete set of PIN transporters translational reporters with fluorescent proteins were generated (PIN1 [56], PIN2 [90], PIN3 [91], PIN4 [89], PIN6 [92,93,94], PIN7 [88], and PIN8 [94,95,96]; Figure 2). In case of PIN5, the translational fusion with the *GUS* reporter was published [92]. Particularly, the PIN1 protein localization in combination with the *DR5* reporter served to predict auxin accumulation as a common modulator for organ formation in many plant developmental processes [56], e.g., embryo development [44,56], defining apical-basal axis in embryo [84,97], lateral root primordia formation [98], primordia development of inflorescence meristem [52], vascular pattern development in leaves [60], leaf shape [99,100], or de novo organ formation from explants [101]. In addition, in the root apical meristem, combined action of PIN1, PIN2, PIN3, PIN4, and PIN7 is considered to establish a local auxin “reflux loop”, thus maintaining the activity of the root apical meristem [88].

### 2.4. Immunolocalisation and In Situ Hybridisation Approaches

A complex expression pattern of *ARFs* and *Aux/IAAs* in the shoot apical meristem was provided by RNA in situ hybridization [62]. Whole-mount in situ hybridisation and immunolocalisation techniques served to detect mRNA and proteins of PIN efflux carriers in *Arabidopsis* seedlings [88,102,103]. Together with GFP reporters, the antibodies against auxin transporters helped to define their cellular localization, particularly anti-PIN1 [104], anti-PIN2 [105], anti-PIN3 [106], and anti-PIN4 [51].

## 3. Direct Methods for Tracking Auxin Distribution

### 3.1. Immunolocalisation of IAA

A high amount effort was also invested to directly visualise IAA by specific antibodies *in planta*. In *Arabidopsis*, the successful use of IAA antibodies confirmed IAA accumulation in accordance to *DR5* reporter in columella initials and the QC region of the mature root and in lateral roots [56,107]. In addition to *Arabidopsis*, immunolocalisation of IAA was applied in several other plant species to monitor auxin levels during development, e.g., in developing peach leaf cells [108], sunflower embryos [109], tobacco embryo [110], maize coleoptile tips [111], or during the adventitious root formation from cotyledon explants of walnut [112]. Nevertheless, even if the IAA visualisation using antibodies can show auxin accumulation in plants, it seems that the immunolocalisation of such small molecule, like IAA, is not a suitable approach on the sub-cellular level [113].

### 3.2. Radiolabelling

#### 3.2.1. Traditional Methods for Studying Polar Auxin Transport in Plants

One of the original methods how to directly track auxin movements in plants employs radioactively labelled molecules of IAA or other natural and synthetic auxins. Different strategies for different purposes in various plant species and cell cultures have been developed in order to investigate the basics of polar auxin transport and its role in plant development [114]. This methodology has been also used for the functional characterisation of auxin transport carriers [115,116]. Although having the advantage of being possibly carried out in any desired mutant background, this approach has certain limitations. Despite the progress in the development of microscale manipulator techniques, the spatial resolution of the method still remains the major limit. The radioactively supplemented source of auxin is applied on plant tissue segments that are covering several cell types. Moreover, tissue-specific dissection of plant organs for scintillation quantification has not been achieved. Thus, the method is not suitable for determination of local auxin changes in specific tissues [26]. The second major limitation is represented by passive diffusion of auxin through cell plasma membranes from the donor source, which may influence the overall outcome of the transport evaluation. For this reason, proper controls have to be performed to minimize the impact of this background process, e.g., simultaneous application of labelled auxin with the compound of similar size and polarity, which is not transportable by the active auxin transport machinery. Also, the treatment with auxin transport inhibitors helps to reveal background diffusion by blocking active transport [115].

The fundamentals of the complex polar auxin distribution in roots and its influence on root elongation and georeaction were laid in 1980’s, when evidences of two-directional IAA transport were exposed—the acropetal transport towards the root apex in stele and basipetal transport from the apex towards the base in the outer root cell layers [117,118,119,120,121]. Auxin is transported basipetally in a single polarity in stems including hypocotyls and inflorescences [122,123,124,125]. In the very first assays, lanolin paste or agar blocks were used as a donors of radiolabelled IAA and the radioactivity was measured in receiver agar blocks in the opposite site of the examined segment [121]. The spatial resolution of this approach was sufficient only for bigger plant species, such as *Zea mays* [117], *Phaseolus coccineus* [118,119,122,123], or *Vicia faba* [120,121,126]. The first attempt to measure direct auxin transport in *Arabidopsis thaliana* was performed by Okada [127], who transferred cut inflorescence segments of *Arabidopsis* into microtubes with a small amount of liquid source of ^14^C-IAA, while measuring radioactivity at the other end of the inflorescence. This study confirmed the basipetal transport of auxin in the plant shoot, and revealed the importance of PIN1 transport carrier in this process as playing a role in proper floral bud formation [127]. For the root polar auxin transport mechanisms, optimised handling of this assay was developed using ^3^H-IAA-supplemented agar cylinders made with a narrow stem transfer pipette to only locally apply ^3^H-IAA to the root tip. By that means it was found that the basipetal auxin transport in agravitropic mutant of *pin2* allele *eir1-1* is altered, while the acropetal auxin transport remains undistinguishable from the wild-type. This experiment demonstrated that the apex-to-base direction of IAA flux is responsible for gravitropic responses in *Arabidopsis* [128]. The measurements of auxin in hypocotyls can be more difficult because of the weak uptake of IAA from the aqueous media into the intact hypocotyl, and therefore it is helpful to dissect the shoot apex and place the agar block on the decapitated site [115].

With these methods auxin movement was measured as the amount of IAA transported between the donor and the receiver site of the plant segment over a defined period of time. It defined an auxin flux, while the quantification of radioactive auxin in several loci with an increasing distance from its source will determine the rate of the auxin transport [115]. This was done by performing the “pulse-chase” assay when the plant tissue is treated for a short time with radioactive auxin, followed by a longer treatment with non-labelled auxin for defined periods of time. The tissue is then cut into segments, and the level of radiolabelled IAA in each segment is quantified [129]. This method helped to determine the differences between transport rates of IAA and IBA in both *Arabidopsis* root and inflorescence tissues. No IBA movement was detected in the inflorescence when compared to basipetal IAA transport at the rate of 13–15 mm per hour. In roots the basipetal transport of both IBA and IAA displayed the same rate of 8–10 mm per hour [129].

All of the protocols and methods of radiolabelled auxin applications for determination fluxes and rates of auxin transport in roots, hypocotyls, and inflorescences are reviewed in Lewis and Muday [115]. Taken together, the measurement of radioactively labelled auxins represents a very sensitive and fast technique for the direct tracking of auxin *in planta.* These methodologies have significantly contributed to the elucidation of the basic principles of the polar auxin transport in different developmental processes [127,128,129,130,131,132]. It also has been crucial for the determination of the functionality of auxin transport carriers responsible for the precise regulation of the auxin polar transport in plants [32,133,134,135,136,137].

#### 3.2.2. Cellular Polar Auxin Transport Matters

The above-mentioned methods that are based on the detection of movement of radioactively labelled auxins provided information about overall auxin polar transport within distinct organs and tissues and its impact on plant morphogenesis. However, suspension-cultured cell lines may represent a sensitive system for evaluation of kinetic parameters of individual auxin transporters, their substrate specificity or the role in promoting and regulating auxin fluxes from and into the cell. Based on the accumulation of radioactivity in the tobacco cell culture, Delbarre et al. [138] published comparative data on two synthetic auxins ^3^H-NAA and ^14^C-2,4-D. This study showed that these two analogues behaved differently across the plasma membrane. 1-NAA appeared to be transported by passive diffusion into the cell, but required carriers for active efflux. On the contrary, 2,4-D required active auxin influx to get into the cell while it was shown as a weak substrate for auxin efflux transporters [138]. Based on these findings, these two molecules are used to dissect these two processes and to study auxin influx and efflux independently. The selective affinities of 2,4-D and 1-NAA to auxin transporters have been later confirmed in *Arabidopsis* suspension-cultured cells [139,140]. Nevertheless, Hošek et al. [141] detected an increased accumulation of 2,4-D in BY-2 tobacco cells after 1-naphthylphthalamic acid (NPA) treatment suggesting its role as a substrate for auxin exporters. Moreover, a proposed mathematical model for 2,4-D transport includes possible passive diffusion contributing to its influx and efflux. It is in concert with previously published evidence demonstrating a contribution of diffusion (influx/efflux) and active efflux to 2,4-D transport in *Nicotiana tabacum* L. cv. Virginia Bright Italia (VBI-0) cells [142,143], and BY-2 over-expressors of the *Arabidopsis* gene *PIN7* [86].

In addition, expressing auxin transporters in heterologous systems helps to overcome certain limitations of this approach. Due to metabolic changes such as conjugation or inactivation of auxin in the plant cell systems, it is hard to determine the precise pool of free auxin to be transported [114]. Moreover, because the regulation of auxin transport is a very complex process that is driven by multiple carriers, which may be functionally redundant, expression of the desired transporters in a non-plant system will separate auxin influx and efflux, and solve the problem of redundancy. So far, several heterologous systems, such as yeast [32,33,86,137,144,145,146,147], mammalian cells [32,33,86,144,146], or oocytes of *Xenopus laevis* [148] have been prepared to evaluate specific roles of desired transport proteins in the auxin transport machinery. However, some substrate specificity, inhibitory sensitivity, and kinetic parameters of heterologously expressed proteins were observed [32,86,146], and have to be kept in mind for further studies in plants.

### 3.3. Fluorescent Labelling

#### 3.3.1. Strategies to Label Plant Hormones

Even though the indirect detection of the auxin action using auxin-sensitive reporters provides a powerful tool, which has been widely exploited for several years to study the modes of auxin distribution, these methods have certain limitations. Firstly, the overall signal output from the reporter expression is an indicator of the presence of auxin, including local biosynthesis and metabolism, to the transport contribution. Likewise, the cells promoting auxin transport are not necessarily sensitive for auxin signalling. Moreover, these reporter transgenes are not available for all of the model species and the introgression of the reporters in mutant lines is time-consuming. Finally, since the regulation of the auxin transport machinery is a very dynamic and complicated process, all of the indirect and invasive methods for auxin detection are no longer sufficient for both temporal and spatial resolution of auxin monitoring. Consequently, the efforts are made to develop microscale techniques to visualise auxin tissue-specific, as well as inter- and intracellular transport in real time [149].

The current conception of studying molecular and structural insights of plant hormone modes of action is based on the interplay between biology and chemistry. Libraries of diverse structural analogues of phytohormones led to discoveries on the relationships between their structure and their biological effect (structure-activity relationship—SAR) [150]. It helped to unravel the essential parts of the molecule responsible for its biological activity from the non-essential moieties, which can be modified for different purposes. This chemical biology approach opened a new field how to study the biological properties of small compounds that are involved in plant growth and development. Employing fluorescent labels that are conjugated with hormone molecules provides very useful tools to visualize their distribution in vivo in real time in all organs and tissues at cellular and sub-cellular levels. They can also help to identify the sites of their perception by creating detectable receptor-ligand complexes [151]. In combination with rapidly developing and very sensitive microscopic imaging techniques, fluorescently labelled phytohormones represent a modern approach with high spatio-temporal resolution to investigate the coordination of their transport, perception and mode of action regulating all the aspects of plant development and responses to various environmental stimuli. Moreover, regarding the usage of fluorescent compounds, no transformation of reporter construct is needed to detect the presence of the hormone. Thus, the determination of its distribution can be elucidated in any chosen plant line [149].

**Figure 3:**
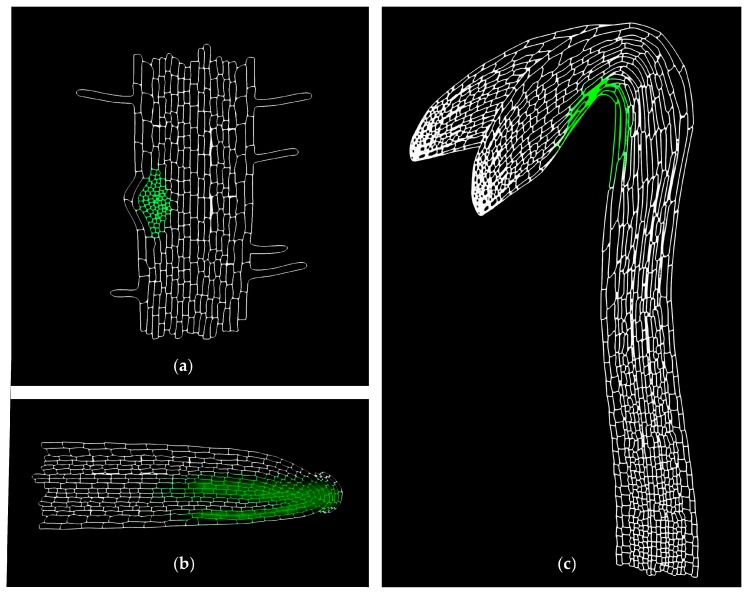
Scheme of tissue-specific localization of fluorescent auxin analogues. The active auxin transport carriers regulate the asymmetric distribution of auxins within different developmental processes. The distribution pattern of fluorescently labelled auxins should mimic the native IAA gradients in specific tissues such as (**a**) lateral root initiation sites, (**b**) the lower side of gravistimulated roots, or (**c**) the concave side of apical hook. Moreover, the non-specific fluorescent signal needs to be investigated, for instance by using a fluorescent analogue non-specific for polar auxin transport machinery. Green color represents localization of auxin analogue labeled with green fluorophore, e.g., NBD (7-nitro-2,1,3-benzoxadiazole).

The synthesis of the fluorescent analogues is preceded by the selection of the optimal structure design. This can be achieved based on the structure-activity relationship information coupled with computational modelling, which provides structural information about the target protein based on its crystal structure. In silico screening of proposed structures with the protein binding site can help to predict the best option of modification when considering the theoretical binding interactions. Nevertheless, the real overall chemical features of the derived molecules influenced by both the used linker and the fluorescent label have to be borne in mind. The position of the labelling site [152], and the character and the length of the linker [153,154,155] play a crucial role in the bioactivity of the new hormone analogues. Also, the choice of the fluorescent probe has to be considered. In general, there are three possible ways how to fluorescently label and visualise the object of interest for imaging: (i) fluorescent proteins; (ii) small organic fluorophores (Figure 3); and, (iii) quantum dots—QDs [156]. Talking about hormones, small bioactive molecules, only the last two approaches can be taken into account. QDs are not very often used in phytohormone field [157,158,159]. Small organic fluorophores are still the most important players with the commonly used fluoresceins (FITC), rhodamines (RITC), coumarins, NBD (7-nitro-2,1,3-benzoxadiazole), and BODIPY (boron-dipyrromethens) dyes. Furthermore, a plethora of their structural analogues covers the whole UV-VIS spectra of emission wavelengths, so one can choose according to their application needs [156]. Because of the distinct pH conditions inside the cells, in apoplast and different organelles, pH sensitivity of the labels has to be taken into account. For example fluorescein is very sensitive to pH changes and gets protonated below pH 7, resulting in significant decline of its fluorescent intensity due to a reconfiguration of the fluorophore’s π-electron system after protonation [160]. On the contrary, BODIPY and Alexa Fluor dyes lack pH-dependent ionizable substituents, making them pH-insensitive alternative to FITC [161,162]. In addition, rhodamine-based labels are more photo- and pH-stabile, but they suffer from bad water solubility [163]. Nevertheless, Alexa Fluor dyes are negatively charged, which may influence the distribution of their conjugates [161].

As indicated above, the tracers together with the linkers significantly differ in chemical and physical properties, and therefore their application may change the behaviour of the tagged molecules, such as solubility, charge, hydrophobicity, or fluorescent intensity, resulting in altered physiological properties, e.g., the speed of uptake, perception, transport dynamics, or metabolism [156]. Hence, before the fluorescent analogues of endogenous hormones can be used as a tool to study molecular insight of their activity, all of the mentioned details should be investigated using in vitro and in vivo bioassays to confirm that the addition of the fluorophore and the linker counterparts does not alter the physiological properties of the hormone. Moreover, the possible enzymatic degradation of the fluorescent construct in living systems has to be considered and elucidated with sensitive methods to (i) minimize misinterpretations of data obtained when using fluorescent hormone analogues; and, to (ii) obtain credible data of the hormone distribution based on the fluorescent pattern. Additionally, since the fluorescent hormone analogues are applied exogenously in non-physiological concentrations, the artificial non-specific fluorescent signal and the real tissue-specific accumulation need to be distinguished properly. For that purpose, negative fluorescent controls that provide a fluorescent signal but are not recognised by auxin transport carriers can be used [164]. If the uneven distribution of the compounds during auxin-related developmental processes is driven by the polar auxin transport, then the fluorescent maxima in the specific tissues can be expected (Figure 3). Negative controls should not exhibit this accumulation.

#### 3.3.2. Up-To-Date Labelling of Auxins

The SAR analysis investigating auxin structural insights revealed only two moieties crucial for its biological activity—system of one or more aromatic rings and carboxyl group side chain [165,166]. The ring structure can be modified significantly, showing a high level of promiscuity of the auxin receptor binding site [63]. Despite the secondary amino group of the indole ring of IAA contributes to the interaction with the receptor by creating hydrogen bonds, it is not needed for the proper binding of auxin into the binding pocket of the receptor [63,167,168]. Unlike the carboxylic group, different positions in the aromatic ring structure can be used for the attachment of fluorescent moeity. The first published fluorescently labeled IAA was used to study the biological activity of humic substances and their possible interactions with the receptor for IAA in carrot cell culture [169]. The conjugation of FITC with both IAA and low molecular weight fraction (LMr) of humic substances revealed a correlation between the fluorescent patterns of FITC-IAA and FITC-LMr on cell membranes of *Daucus carota*, suggesting that IAA and LMr fractions bind the receptor in the same way [169]. However, neither the structure of the FITC-IAA conjugate, nor its stability in carrot cells were discussed, which makes the observed results hard to interpret. More recently, Sokołowska et al. [170] have presented new fluorescent conjugates of RITC and FITC fluorophores with IAA via the secondary amino group of the indole ring. These compounds have been shown to retain auxin-like biological activity and its distribution pattern has been driven by auxin transport system. Even though the used dyes themselves are thought to be transported differently (RITC by apoplastic, FITC by symplastic transport), the fluorescent compounds exhibited a similar distribution pattern to the one of free auxin [170]. Nevertheless, a mass spectrometry (MS) analysis of the tested compounds revealed the cleavage of the conjugates with a release of fluorophore from IAA. The fragmentation is discussed to take place during the MS analysis. But, the fact that it may be due to enzymatic degradation *in planta* still needs to be taken into consideration while interpreting the data based on the biological activity and of the observed fluorescent pattern. To our knowledge, the last published attempt to produce fluorescently labelled auxin was performed by coupling of two different auxin compounds—IAA and NAA—with NBD tag [164]. Based on the previous research of alkoxy-auxin analogues as competitive inhibitors of auxin transporters [171], the new fluorescent analogues were synthetised with NBD introduced on 5-hydroxy-IAA and 7-hydroxy-NAA. These compounds were designed to be active for auxin transport machinery, but neither for the auxin signalling TIR1/AFB pathway, nor for the GH3-dependent metabolism pathway. Both NBD-IAA and NBD-NAA have been shown to exhibit the pattern of distribution similar to the DR5 pattern in free auxin-treated roots. Moreover, the presence of NBD-auxins in endoplasmic reticulum of cultured cells confirmed that such compounds enable tracking auxin gradients with high spatio-temporal resolution on the subcellular level [156,164].

### 3.4. Microelectrodes

Another method for direct non-invasive monitoring of auxin fluxes in vivo employs IAA-selective microelectrodes [172,173]. Organogenesis and reactions of plants to environmental stimuli are driven by dynamic auxin transport generating auxin gradients in specific tissues [174]. This uneven auxin distribution creates an electrical potential across the organ [175,176], where the side with higher auxin concentration is considered as positive (secreting more H^+^ ions) compared to the side with lower auxin levels. These electrical potentials can be surface-measured using microelectrodes [124]. To be used for continuous recording of auxin transport and the quantification of the local IAA levels, the electrochemical sensors must display a high selectivity for IAA, sensitivity, fast response times, and calibration stability. Mancuso et al. [172] used a platinum electrode with surface-immobilised multiwalled carbon nanotubes (MWNTs) and with a small planar sensing tip for good spatial resolution in combination with a self-referencing electrode to measure auxin transport in root apices of *Zea mays*. Even though the usage of MWNTs enhanced the method sensitivity when compared to a bare platinum electrode, the detection limit of only 0.1 µM IAA was achieved, and thus an exogenous application of IAA had to be performed. Nevertheless, this method was presented as a useful approach for the direct determination of IAA in root samples, direct measurements of its local concentrations, and measurement of IAA fluxes in different positions along the maize root. The study demonstrated that the most intensive influx rate is in the transition zone. This peak in flux (expressed in fmol·cm^−2^·s^−1^) at 1.0–1.5 mm above the root apex corresponds to the auxin reflux loop model [88]. Moreover, the application of auxin uptake inhibitors significantly decreased the influx of IAA into the cells, resulting in a drop of the flux peak for this zone [172]. Although this microelectrode method is applicable only on cells at the root surface or on a thin cell layer, it appears to be a valuable tool for detecting auxin fluxes and has helped to discover and characterise several auxin transport mutants and inhibitors of auxin transport carriers [146,177,178,179,180,181]. This method has been improved by using platinum black and carbon nanotube surface modifications, which helped to increase signal-to-noise ratio [173]. Together with better signal processing and data integration, it enabled directly and non-invasively measuring endogenous IAA transport parameters, with no external source of IAA needed. This enhanced method was used to determine the differences in IAA movements in roots of wild-type maize and auxin transport mutant maize [147,182]. The most intense transport of endogenous IAA was detected in the distal elongation zone of maize roots. Expectedly, the flux of auxin in transport mutant was significantly reduced [173], which correlates with the effect observed in *Arabidopsis thaliana* that is caused by the loss of function mutant of orthologue transporter in *Arabidopsis* [32]. Furthermore, the detection of inhibition of both IAA efflux and influx after treatment with auxin transport inhibitors points out the potential of self-referencing microsensors as a valuable approach for in vivo non-invasive monitoring of IAA transport despite it is still limited to the root surface layers and epidermal cell [173].

## 4. New Valuable Tools to Visualize Auxin Metabolites

The regulation of bioactive auxin levels is complex, and cell- and tissue-specific metabolic profiling can help to answer many questions about local IAA biosynthesis and degradation, as well as auxin transport and the formation of auxin gradients. This short summary does not present the whole picture of auxin profiling methods. For more recent and specific overviews of novel bioanalytical approaches, including the advances of mass spectrometry (MS) and biosensors, we refer the reader to other publications [183,184,185,186].

### 4.1. Cell-Type Specific Mass Spectrometric Analysis

**Figure 4:**
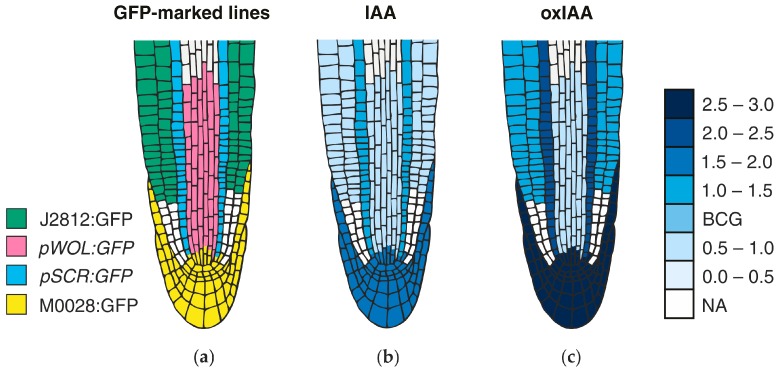
Auxin distribution map within the *Arabidopsis* root tip. (**a**) The data presented in the map was derived from four green fluorescent protein (GFP)-tagged *Arabidopsis* lines (J2812:GFP, *pWOL:GFP*, *pSCR:GFP* and M0028:GFP), covering almost all of the different cell types of the root apex. (**b**,**c**) Roots from eight-day-old *Arabidopsis* seedlings were protoplasted and sorted using FACS, and the concentrations of IAA (**b**) and oxIAA (**c**) were quantified in the separated GFP-expressing cell populations using LC-MS/MS. Cell type-specific concentrations of both auxins were calculated in fmol per 100,000 isolated GFP-expressing protoplasts and then normalised to the non-GFP-expressing reference population for each GFP cell line. “NA” represents cell populations that were not analysed; “BCG” means the background level. The maps were constructed based on the IAA and oxIAA levels published in [11].

MS-based quantitative measurement of auxins on a tissue and at a cellular level is a difficult task, not only due to extremely low concentrations (fmol–pmol/g of fresh weight), but also due to the presence of interfering substances in the plant matrix (e.g., pigments, lipids, phenolic compounds, or proteins) [187]. Together with chemical/thermal/light lability and enzymatic/oxidative degradation of auxins during the extraction and isolation steps, accurate and precise determination is highly challenging [184]. Recent technical advances in analytical methods helped to detect more IAA metabolites (precursors, catabolites, and conjugates) in one sample, and thus to obtain information about the overall pattern of auxin metabolome. Gas chromatography (GC) and liquid chromatography (LC), coupled to tandem mass spectrometry (MS/MS) are often used in the analysis of the most known auxin metabolites, the substances with very different physicochemical properties [188,189]. Several MS-based measurements confirmed the auxin gradients in meristematic tissue sections, such as cambial meristem [190,191] and isolated cell types of the *Arabidopsis* root apical meristem [11,113] (Figure 4). Moreover, a single-cell-resolution analysis of IAA and other phytohormone metabolites in the *Arabidopsis* guard cell protoplasts has been recently published [192].

Cryo-sectioning is a popular method of minute plant tissue sampling, which often provides sufficient cell-type-specific resolution for hormone profiling. For example, the IAA distribution in 30-µm tangential sections that were obtained across the cambial region was measured by GC-MS [190]. To connect the hormone distributions to the status of hormonal signalling and homeostasis, a genome-wide gene expression profiling at a high resolution across the cambial zone were performed [191]. These results suggest that most of the auxin response genes showed maximal expression in the middle of the cambial zone, coinciding with the peak in auxin content.

Another possible high-resolution cell-type specific method is based on the auxin quantification in root cell populations that are sorted by fluorescence-activated cell sorting (FACS). This approach enables the recognition of isolated protoplasts of similar size and granularity, followed by their sorting into homogenous cell-type groups according to the presence or absence of internal fluorochromes (e.g., GFP). In isolated protoplasts that are derived from *Arabidopsis* mutant lines expressing GFP in specific root cell types, the presence of IAA concentration gradients within the root tip with a distinct maximum in the organizing QC of the root apex has been confirmed [113]. Interestingly, cell type-specific auxin measurements do not effectively match DR5 expression in the root apex, however, graded auxin response more closely fits measured auxin concentrations [193]. The found auxin distribution also confirms the hypothesis of different polarisation of PIN proteins at the root apex, resulting in auxin accumulation in the root cap [194]. In Figure 4, the IAA distribution map shows a concentration maximum in the lateral root cap, columella, columella initials, and QC cells. A similar gradient was also found at concentration levels of oxIAA, the primary auxin catabolite formed in the *Arabidopsis* roots [11]. Its origin at the cellular level contributes, in addition to active transport, to maintaining the correct IAA minima/maxima ratios that are necessary for proper root growth and development.

### 4.2. Auxin Monitoring by Solid-State Biosensors

As mentioned above, hormonal signalling reporters and sensors are preferred for in vivo and real-time detection of auxin in living tissues [195]; nevertheless, other biosensors also offer real-time and in vivo quantitation of auxin [186]. Generally, a biosensor is a sensitive analytical device combining a biological component module for the analyte’s recognition with a physicochemical detector, which converts a biological response (e.g., immunochemical or electrochemical reactions) into a signal that can be captured and interrogated [183]. Several reviews have discussed the applications of solid-state biosensors that are used for ex vivo and in vivo monitoring of auxin metabolites [183,185,186]. For example, immunosensors designed for IAA detection can be classified based on the type of the detector: (i) electrochemical [196,197]; (ii) photoelectrochemical [198]; and, (iii) piezoelectric [199]. Other types of biosensors make use of molecular imprinted materials (MIPs), which also selectively recognize a template molecule. Several examples of the MIPs application to auxin quantification can be found in the literature [200,201]. However, affinity-based sensors often required an analyte extraction from plant tissues and one or more steps of pre-concentration. A non-enzymatic electrochemical biosensor system that is based on the direct oxidation of IAA by a graphite paste electrode was also introduced [202], and then modified to carbon nanotube-coated platinum electrodes [203]. Moreover, Mancuso et al. [172] and McLamore et al. [173] used a self-referencing vibrating microelectrode technique for the study of auxin fluxes in root apexes (for more details see Section 3).

In summary, the solid-state biosensors, together with development of genetically encoded reporters and sensors and advances in fluorescent labelling, facilitate the study of auxin signalling and distribution in living intact plants. Several bioanalytical approaches, such as FACS and LC-MS/MS methods, can be equally used for cell-specific analysis of auxins, and thus provides ideas about the coordination of plant hormone metabolism and transport, and the regulation of core signalling component expression.

## 5. Future Prospects

Diverse plant developmental events that are triggered by auxin trafficking, redistribution, and tissue-specific accumulation as a response to ambient conditions represent very dynamic and highly regulated processes. Moreover, the microenvironment in plant tissues is very complex and two neighbouring cells can be in a different state of development, and thus have a distinct function. For those reasons, claims on spatial and temporal resolutions of detecting techniques are increasing. Therefore, the application of mass spectrometry imaging (MSI) and living single-cell MS analysis could soon provide a powerful tool for studying of auxin distribution, even though it is still limited for hormone profiling [186]. Moreover, very little is known about extra- and intracellular distributions of auxins and their metabolites, as well as their levels in individual cell compartments. Separation of organelles for auxin profiling was recently carried out by porous-specific filtration (e.g., gradual separation of chloroplasts and mitochondria [204]) or density-based fractionation (e.g., chloroplasts separation in percol solution [205]). However, a detailed organelle-specific analysis of auxin levels is still lacking. Therefore, we are looking forward to developing new analytical methods and periods of innovative approaches to work at the intracellular level.

To detect and monitor auxin distribution with sufficient spatial and temporal resolution in minimal invasive manner, improvement of above-mentioned methods employing genetic biosensors, as well as novel approaches of live imaging to capture extra- and intracellular hormone dynamics are also demanded. Aside from the development of new expression reporters with high selectivity for auxin molecules responding rapidly to physiological levels of hormones in linear manner so that the response can be quantified [206], new genetically encoded biosensors for the quantitative distribution of biomolecules based on (i) fluorescent translocation sensors; (ii) fluorescence-intensity-based nanosensors; and, (iii) Förster resonance energy transfer (FRET)-based nanosensors are on the rise [207]. Also, the preparation of new fluorescent auxin analogues, which would display biological activity, auxin transporters-dependent distribution, and enzymatic stability, remains a challenging issue. The rapid progress of different microscopic imaging techniques [208] goes hand in hand with inventions of devices allowing for the long-term monitoring of plant growth in vertical position to maintain physiological growing conditions [67,209,210,211,212]. Moreover, employing of microfluidic perfusion system that is controlled by micromechanical valves provides precise and fast control and modulation of the plant environment when reversible delivery of the chemicals of interest is enabled [213,214]. This set of devices, together, in combination with rapid-response and sensitive genetic reporters of auxin action or reliable fluorescent auxin derivative treatment could offer a powerful method to visualize in vivo auxin distribution with real time resolution on all organs and tissues, at the cellular and subcellular levels. In addition, label-free imaging techniques, which have been used for metabolic imaging of high abundant molecules in mammalian cells, such as lipids monitored with coherent anti-Stokes Raman scaterring (CARS) microscopy [215] or ω-3 fatty acids by stimulated Raman scattering (SRS) [216], may soon achieve adequate sensitivity for selective monitoring of plant hormones without any needs of indirect visualization or structure modification, which would enable to track their distribution in different processes in the most natural manner.
